# Supplementary material for: Scarcity of scale-free topology is universal across biochemical networks
Source: Sci Rep. 2021 Mar 22;11:6542. doi: 10.1038/s41598-021-85903-1 (PMC7985396; doi:10.1038/s41598-021-85903-1)
Supplement: Supplementary file 1 — Supplementary Information. [file 41598_2021_85903_MOESM1_ESM.pdf]

Scarcity of scale-free topology is universal across biochemical networks

Supplementary Information

Harrison B. Smith [1,2], Hyunju Kim [1,3,4], Sara I. Walker [1,3,4,5*]

1 School of Earth and Space Exploration, Arizona State University, Tempe, AZ, USA
2 Current affiliation: Earth-Life Science Institute, Tokyo Institute of Technology, Meguro-ku, Tokyo, JP
3 Beyond Center for Fundamental Concepts in Science, Arizona State University, Tempe, AZ, USA
4 ASU–SFI Center for Biosocial Complex Systems, Arizona State University, Tempe, AZ, USA
5 Santa Fe Institute, Santa Fe NM

*** sara.i.walker@asu.edu**

Supporting Information

S1 Fig

How alternative distributions compare to the powerlaw across each network projection type. The proportion of network projections, across all datasets, that favor either the power-law distribution (1.0), an alternative distribution (-1.0), or are inconclusive (0.0). Each row shows a different network projection type. Each column is a different distribution with which the power-law is being compared to. From left to right is the exponential; log-normal; stretched exponential; and power-law with cutoff. Dashed line is constant at proportion = 0.5 across all subplots. Red bars indicate individual-level networks, and blue bars indicate ecosystem-level networks.

S2 Fig

Correlations between network projections which meet scale-free criteria. Correlation matrix heatmaps show type how different types of network projections correlate in their proportions of networks which meet some scale-free criteria. Rows are for each of the different scale-free criteria (p-value, n_{tail} and α), and columns are for individual and ecosystem-level networks. Heatmaps show the correlation between values for each projection type, where the values are of the proportion of networks which meet the scale-free threshold criteria of: $p \geq 0.1$ (top row); $n_{tail} \geq 50$ (center row); $2 < \alpha < 3$ (bottom row). Values from projections of a network's LCC and entire graph are highly correlated. All types of unipartite compound networks tend to be correlated. Values across many other projection types are barely correlated for the p-value and n_{tail} criteria. Ecosystems tend to show more correlation, across all projection types, than individuals.

S3 Fig

Predicting individuals and ecosystems from degree distribution data using multinomial regression. Each subplot shows the accuracy of using a particular network or statistical measure to predict whether that network data came from an biological individual or ecosystem. The subplots in the right column are the accuracy of using a measure after being normalized to network size. Unsurprisingly, network size is by far the best way to accurately predict whether data comes from an individual or ecosystem (left blue star). Once normalized to size, whether or not a degree distribution favors an exponential fit compared to a power-law fit becomes a decent predictor (right blue star). Subplots measures are: power-law alpha value; log-likelihood result from power-law vs. exponential; log-likelihood result from power-law vs. log-normal; log-likelihood result from power-law vs. power-law with exponential cutoff; log-likelihood result from power-law vs. stretched exponential; the log-likelihood of the power-law model; the network mean degree; network node size; degree distribution tail size; network edge size; cutoff degree value for network tail. Prediction accuracy is random if $\leq 50\%$, Fair if $> 50\%$, and Good if $> 75\%$.

S1 Table

Random forest accuracy by network projection type. The predictors used in the random forest are the same predictors used in the multinomial regression: power-law alpha value; log-likelihood result from power-law vs. exponential; log-likelihood result from power-law vs. log-normal; log-likelihood result from power-law vs. power-law with exponential cutoff; log-likelihood result from power-law vs. stretched exponential; the

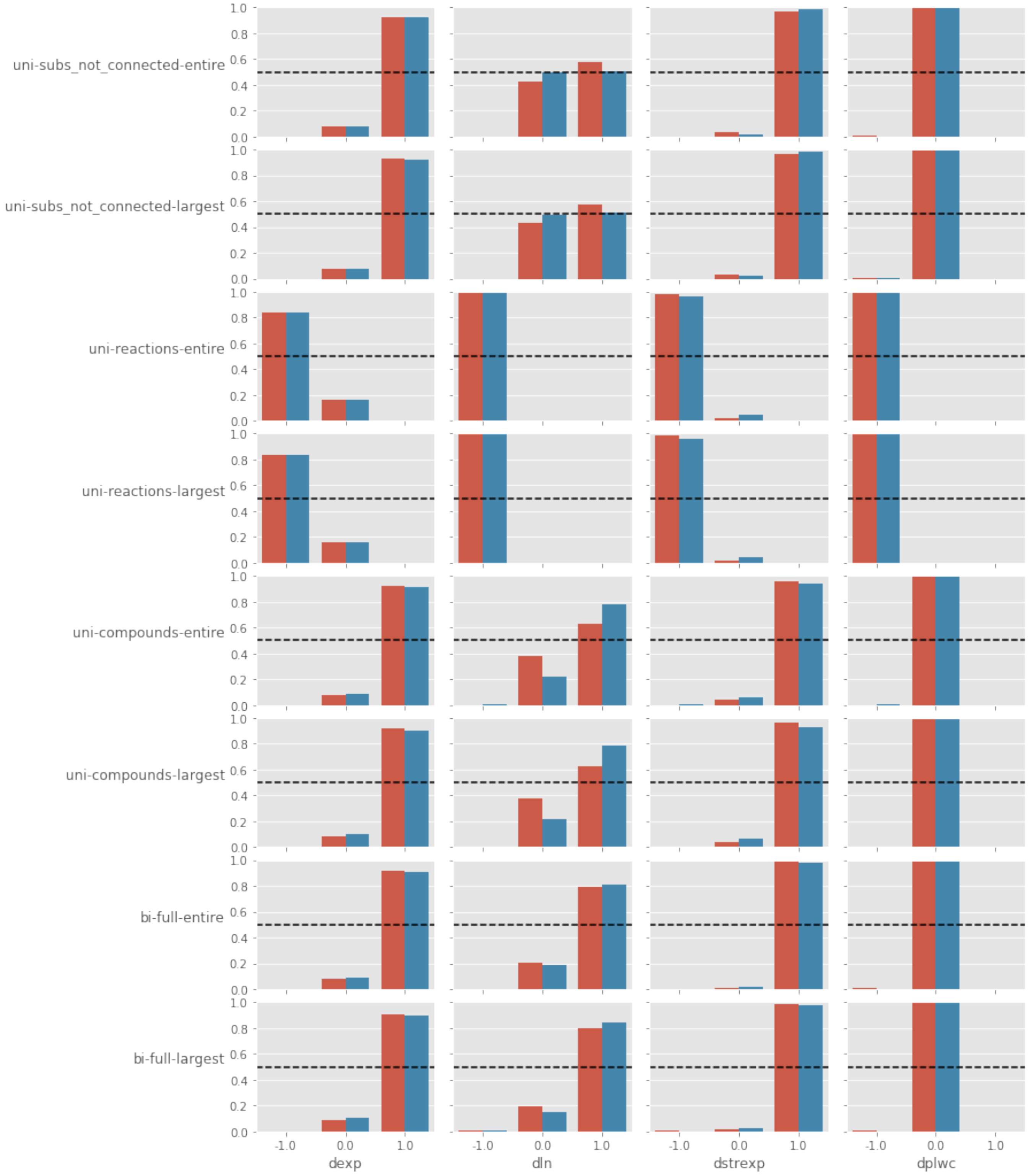

Figure S1. How alternative distributions compare to the powerlaw across each network projection type. The proportion of network projections, across all datasets, that favor either the power-law distribution (1.0), an alternative distribution (-1.0), or are inconclusive (0.0). Each row shows a different network projection type. Each column is a different distribution with which the power-law is being compared to. From left to right is the exponential; log-normal; stretched exponential; and power-law with cutoff. Dashed line is constant at proportion = 0.5 across all subplots. Red bars indicate individual-level networks, and blue bars indicate ecosystem-level networks.

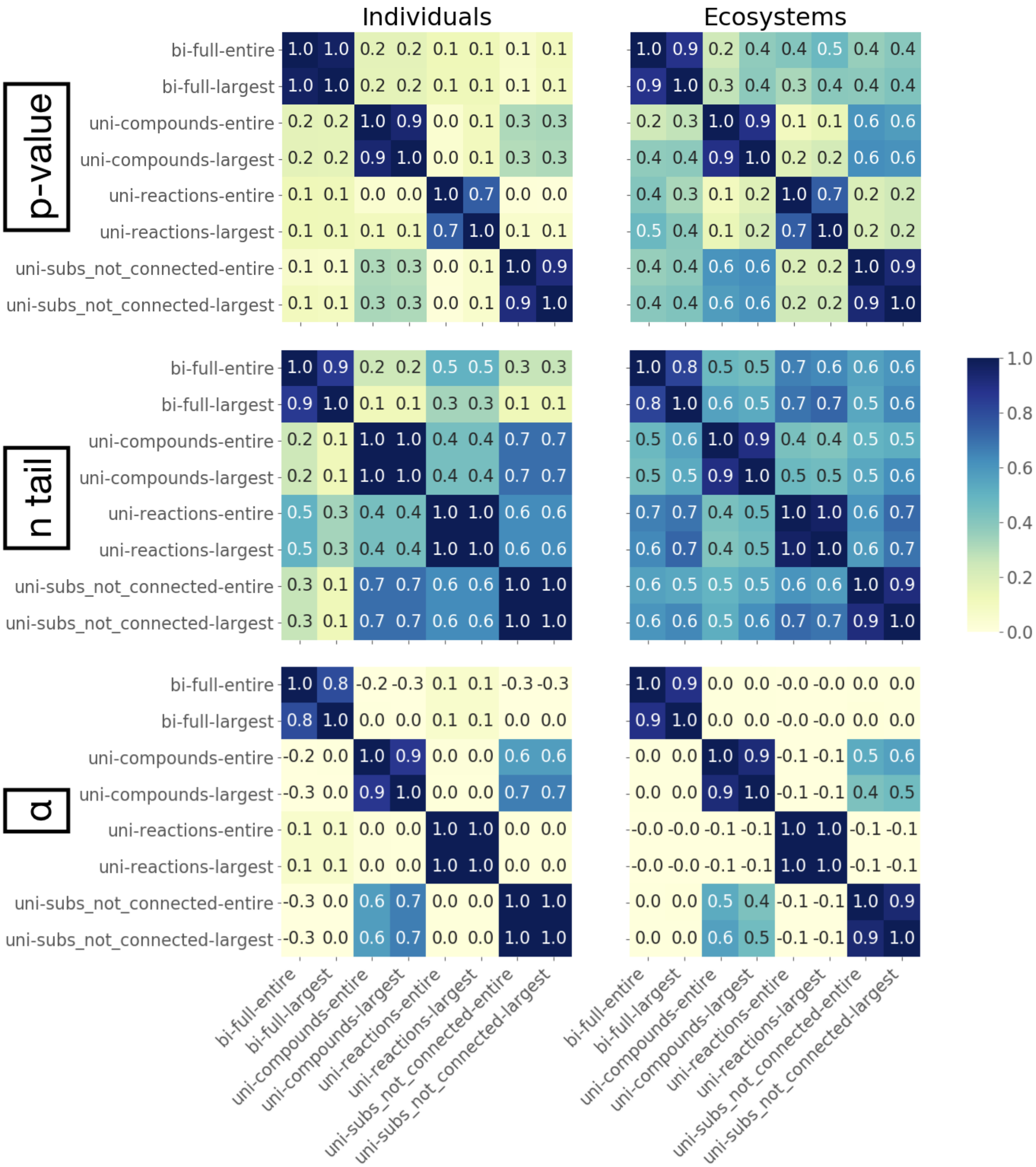

Figure S2. Correlations between network projections which meet scale-free criteria. Correlation matrix heatmaps show type how different types of network projections correlate in their proportions of networks which meet some scale-free criteria. Rows are for each of the different scale-free criteria (p-value, n_{tail} and α), and columns are for individual and ecosystem-level networks. Heatmaps show the correlation between values for each projection type, where the values are of the proportion of networks which meet the scale-free threshold criteria of: $p \geq 0.1$ (top row); $n_{tail} \geq 50$ (center row); $2 < \alpha < 3$ (bottom row). Values from projections of a network's LCC and entire graph are highly correlated. All types of unipartite compound networks tend to be correlated. Values across many other projection types are barely correlated for the p-value and n_{tail} criteria. Ecosystems tend to show more correlation, across all projection types, than individuals.

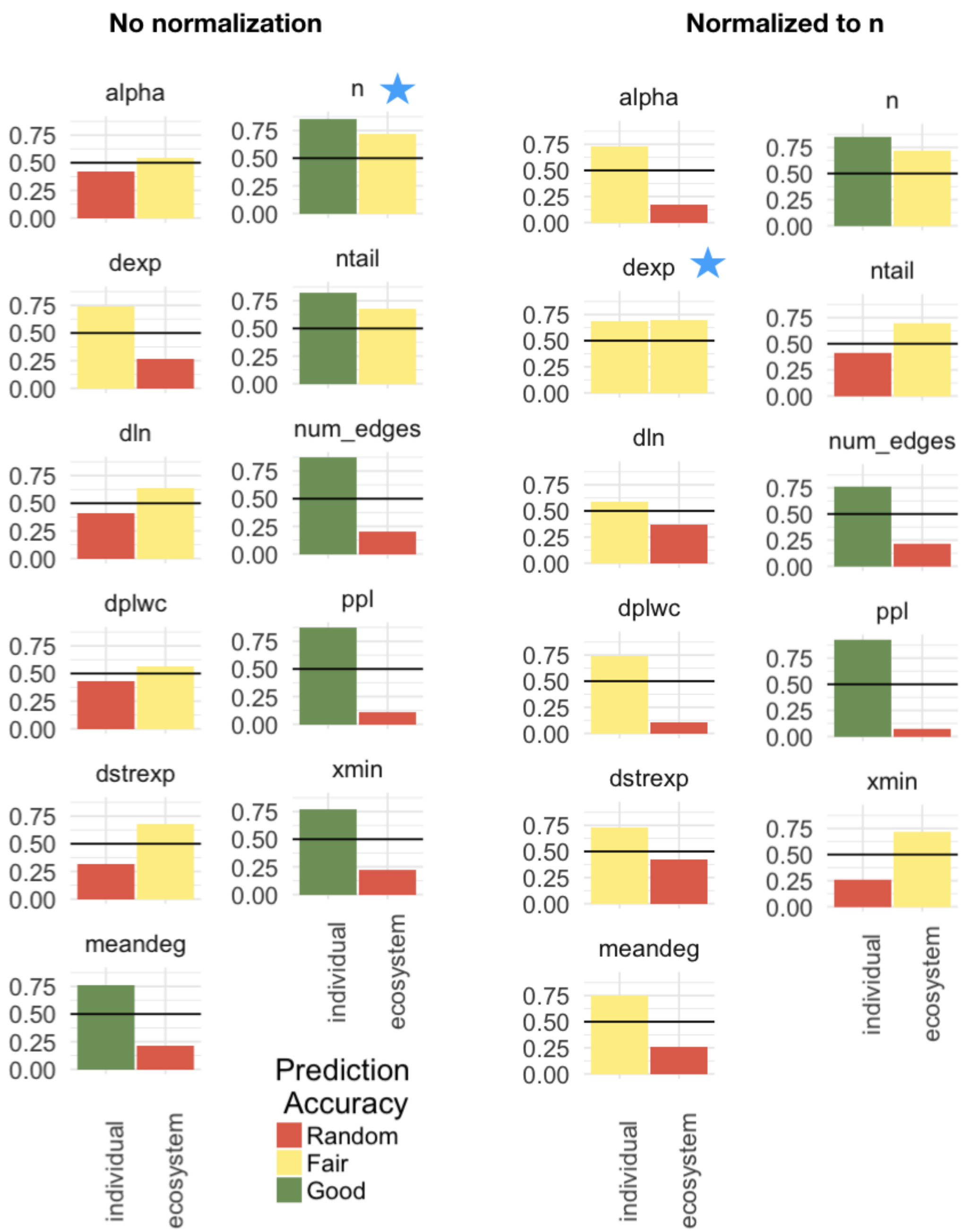

Figure S3. Predicting individuals and ecosystems from degree distribution data using multinomial regression. Each subplot shows the accuracy of using a particular network or statistical measure to predict whether that network data came from an biological individual or ecosystem. The subplots in the right column are the accuracy of using a measure after being normalized to network size. Unsurprisingly, network size is by far the best way to accurately predict whether data comes from an individual or ecosystem (left blue star). Once normalized to size, whether or not a degree distribution favors an exponential fit compared to a power-law fit becomes a decent predictor (right blue star). Subplots measures are: power-law alpha value; log-likelihood result from power-law vs. exponential; log-likelihood result from power-law vs. log-normal; log-likelihood result from power-law vs. power-law with exponential cutoff; log-likelihood result from power-law vs. stretched exponential; the network mean degree; network node size; degree distribution tail size; network edge size; the p-value of the goodness-of-fit test for the power-law model; cutoff degree value for network tail. Prediction accuracy is random if $\leq 50\%$, Fair if $> 50\%$, and Good if $> 75\%$.

network mean degree; network node size; degree distribution tail size; network edge size; the p-value of the goodness-of-fit test for the power-law model; cutoff degree value for network tail. See methods for description of network projection types.

Table S1. Random forest accuracy by network projection type.

Network projection type	Prediction accuracy (%)		OOB error (%)
	Ecosystem	Individual	
**bi-full-entire**	75.58	93.75	13.83
**bi-full-largest**	75.45	94.83	13.29
**uni-compounds-entire**	81.44	93.76	11.36
**uni-compounds-largest**	80.81	93.67	11.79
**uni-reactions-entire**	80.95	93.67	11.36
**uni-reactions-largest**	80.40	93.08	12.33
**uni-subs_not_connected-entire**	76.12	92.93	13.93
**uni-subs_not_connected-largest**	77.47	91.64	14.36

The predictors used in the random forest are the same predictors used in the multinomial regression: power-law alpha value; log-likelihood result from power-law vs. exponential; log-likelihood result from power-law vs. log-normal; log-likelihood result from power-law vs. power-law with exponential cutoff; log-likelihood result from power-law vs. stretched exponential; the network mean degree; network node size; degree distribution tail size; network edge size; the p-value of the goodness-of-fit test for the power-law model; cutoff degree value for network tail. See methods for description of network projection types.
